# Identification of high-risk patients for ADR induced by traditional Chinese medicine injection: a nested case-control study

**DOI:** 10.1038/s41598-019-53267-2

**Published:** 2019-11-13

**Authors:** Cheng Jiang, Jie Shen, Dan Shou, Nani Wang, Jing Jing, Guodi Zhang, Jing Gu, Yunlong Tian, Caihua Sun, Jiaqi He, Jiaqi Ma, Xiaojun Wang, Gonghua Li

**Affiliations:** 1 Department of pharmacy, Tongde Hospital of Zhejiang Province, Hangzhou, China; 2 Department of pharmacy, Zhejiang Province Hospital of Traditional Chinese Medicine, Hangzhou, China; 3 Department of Medicine, Zhejiang Academy of Traditional Chinese Medicine, Hangzhou, China; 4 Department of oncology, Tongde Hospital of Zhejiang Province, Hangzhou, China; 5 Department of endocrinology, Tongde Hospital of Zhejiang Province, Hangzhou, China; 6 College of chemical engineering, Zhejiang University of Technology, Hangzhou, China

**Keywords:** Disease prevention, Epidemiology, Risk factors

## Abstract

The adverse drug reaction (ADR) of traditional Chinese medicine injection (TCMI) has become one of the major concerns of public health in China. There are significant advantages for developing methods to improve the use of TCMI in routine clinical practice. The method of predicting TCMI-induced ADR was illustrated using a nested case-control study in 123 cases and 123 controls. The partial least squares regression (PLSR) models, which mapped the influence of basic characteristics and routine examinations to ADR, were established to predict the risk of ADR. The software was devised to provide an easy-to-use tool for clinic application. The effectiveness of the method was evaluated through its application to new patients with 95.7% accuracy of cases and 91.3% accuracy of controls. By using the method, the patients at high-risk could be conveniently, efficiently and economically recognized without any extra financial burden for additional examination. This study provides a novel insight into individualized management of the patients who will use TCMI.

## Introduction

Adverse drug reaction (ADR) is appreciably harmful reaction caused by an intervention related to drugs and may affect a number of organ systems in the body. The patients may suffer from physical and mental damage due to increased hospitalization, prolongation of treatments or additional clinical examinations. Some ADR can cause severe disorders and even be life-threatening. ADR also lays a huge financial burden on patients and healthcare services. Thus, for both patients and healthcare perspective, there are significant advantages for developing methods to assist treatment decisions in routine clinical practice by predicting the individual’s likelihood of ADR.

Traditional Chinese medicine injection (TCMI) is produced under modern scientific and technological theory to extract and purify the effective constituents from herbs or decoction pieces. It plays an important role in treating severe and acute disease in China. It had been estimated that the annual sales of TCMI have reached more than 30 billion RMB and accounted for one third of all traditional Chinese medicine sales^1^. However, the incidence of ADR induced by TCMI is obviously higher than any other dosage forms of traditional Chinese medicine. According to the survey of ADR case reports, the ADR due to TCMI accounted for more than 40% of all ADR caused by traditional Chinese medicine^2,3^. Moreover, the incidence of severe ADR induced by TCMI was 86.7% among all severe cases induced by traditional Chinese medicine. So far, the ADR of TCMI has become one of the major concerns of public health in China. It is of great importance for developing a simple, robust and reliable method to prognosticate the risk of ADR for individualized management of patients who will use TCMI.

In the case of prediction model, symptom may occur in the future. This is a crucial distinction differ from the case of diagnostic model, in which symptom has already occurred. Therefore, the development of risk prediction model needs large numbers of patients submit to extensive baseline measurements. Clinical trial and real-world evidence are the most two common ways to get baseline measurement. Compared with traditional clinical trial, the real-world evidence has the potential to reflect the patients’ individual factors efficiently, saving time and money while yielding answers relevant to broader populations of patients than would be possible in a specialized research environment^4^. The on-line electronic medical records system stores a large amount of real-world clinical information of patients undergoing hospitalization including basic characteristics and routine examinations. In most hospitals, the basic characteristics are recorded by experienced medical workers and the baseline routine examinations are operated strictly compliance with specifications. Therefore, the availability, accuracy and reliability of clinical information can be guaranteed. For all the above reasons, if prediction model is successfully established based on clinical information, the risk of TCMI-induced ADR can be easily predicted in advance. Nevertheless, no available model for predicting the risk of ADR induced by TCMI based on clinical information has been reported to the best of our knowledge.

Up to now, the clinical information including basic characteristics and routine examinations has been applied to evaluate the physiological and pathological state of individual patient in clinic for decades. Some basic characteristics have been demonstrated to be closely associated with many diseases. For instance, smoking history and hypertension are well known risk factors for lung cancer^5^ and intracerebral hemorrhage^6^. Several studies have found disease biomarkers from the routine examinations, including D-dimer^6^, serum copper^7^, serum calcium^8^, triglyceride glucose, hemoglobin glycation^9^, platelet, neutrophil and lymphocyte^10,11^. Moreover, the risk prediction models based on clinical information have been established in many fields of modern medicine, such as cardiovascular disease and cancer. For example, the prediction model for 10-year risk of cardiovascular disease has been successfully established using the patient’s age, sex, smoking history, diabetes status, systolic blood pressure, total and high-density lipoprotein cholesterol^12^. Accordingly, the clinical information may also have potential associations with the risk of TCMI-induced ADR and present opportunities to create risk prediction model. It definitely seems worth a try to clarify the associations between clinical information and TCMI-induced ADR.

In this study, the method of predicting TCMI-induced ADR was illustrated with the application of real-world practice. The partial least squares regression (PLSR) models, which mapped the influence of basic characteristics and routine examinations to ADR, were established to predict the risk of ADR. The software was further devised to provide an easy-to-use tool for clinic application. By using this method, the patients at high-risk could be conveniently, efficiently and economically recognized without any extra financial burden for additional examination. This study provides a novel insight into individualized management of patients who will use TCMI.

## Results

### Description of cases and controls

**Figure 1:**
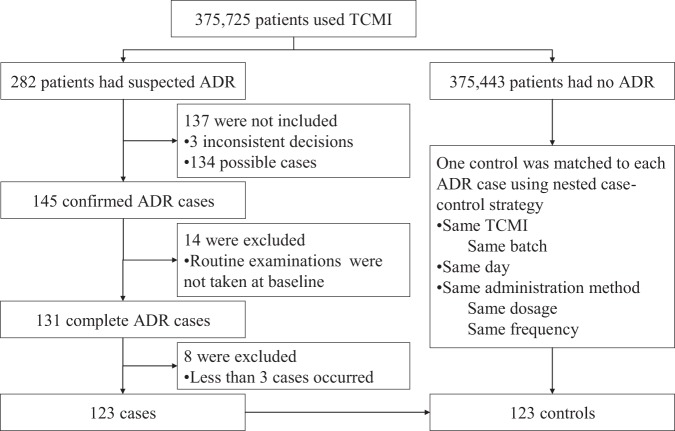
Flowchart of participant selection.

From January 2013 to December 2018, there was a total of 375,725 patients used TCMI, among which 282 had suspected ADR. 3 cases had inconsistent decisions. 279 cases with consistent decision were grouped as 4 “definite”, 141 “probable” and 134 “possible”. On account of the multi-factors characteristic of ADR, only 145 confirmed cases (4 definite and 141 probable) were involved. In order to eliminate the risk that the examinations results might be affected by medications or treatments, 14 cases were excluded since the routine examinations were not taken at baseline. For statistical considerations, 8 cases were excluded since less than 3 cases of a TCMI occurred. A total of 123 ADR cases met the selection criteria for the study. The herbal species and major active compounds of each TCMI in included cases are outlined in Table s1 in the Supporting Information. In 123 cases, the most manifestations of ADR were systemic reaction. The ADR severity for each case was classified according to World Health Organization guidelines. Most of the cases were class IV. The ADR manifestations and seriousness in included cases are outlined in Tables s2–s3 in the Supporting Information. Finally, 100 cases and 100 controls were utilized as calibration cohort, while 23 cases and 23 controls were utilized as external validation cohort. The flowchart of participant selection is shown in Fig. 1.

### Development of PLSR model

**Figure 2:**
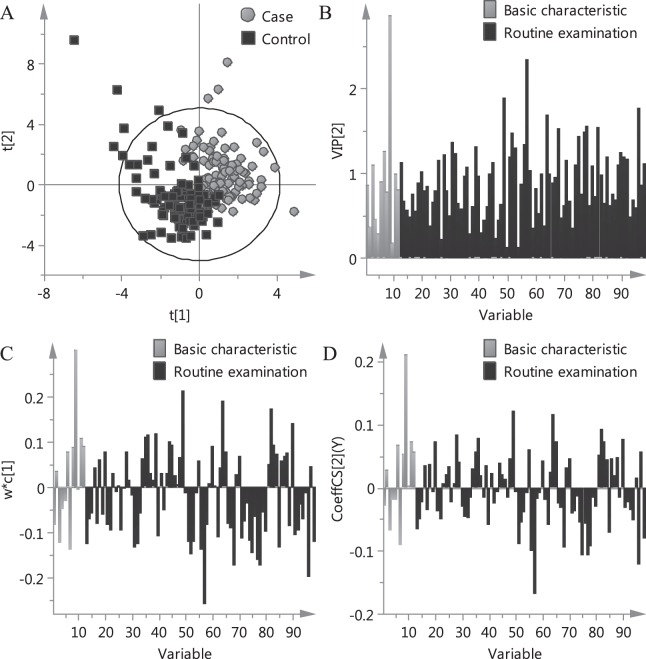
Plots of PLSR model. (**A**) Scores plot displayed how the X observations were situated with respect to each other. Score of first component explained the largest variation of the X space, followed by score of second component. (**B**) VIP plot displayed the importance of the variables both to explain X variables and to correlate to ADR. (**C**) Loading weights plot summarized the relation between the X variables and the ADR. (**D**) Regression coefficients plot reflected how strongly ADR was correlated to the systematic part of each X-variable.

The PLSR model was established based on basic characteristics and routine examinations combined with data fusion strategy. The scores plot of PLSR model is shown in Fig. 2A. The cases and controls tended to cluster in different regions of the scores plot, indicating that there were different characteristics between the two groups.

The variable importance for the projection (VIP) values, which summarize the importance of the variables both to explain *X* and to correlate to *Y*, were used to screen the important variables. The w*c [1] loading weights and regression coefficients were applied to investigate the relationship between *X* and *Y*. The VIP plot, loading weights plot and regression coefficients plot are shown in Fig. 2B–D. The top ten variables with the highest VIP values are shown in Table 1. According to the PLSR results, history of trauma surgery was the most important risk factor for ADR. Cases exhibited higher values of platelet distribution width, high-density lipoprotein and globulin. In addition, lower values of albumin/globulin, activated partial thromboplastin time, urea, serum creatinine, serum sodium and neutrophil count in cases than in controls were also pronounced.

**Table 1:** PLSR and univariate data analysis results of ten typical variables.

Variable Names	Variable NO.	Cases (n = 100)	Controls (n = 100)	Unit	PLSR results			Univariate data analysis results	
					VIP	Loading weights	Coefficients	Test methods	*P* value
History of trauma surgery	9	49	17	Person	2.856	0.304	0.210	χ2 test	<0.001
Albumin/globulin	57	1.5 ± 0.3	1.7 ± 0.4	/	2.340	−0.258	−0.167	*t* test	<0.001
Platelet distribution width	49	16.1 ± 1.5	15.1 ± 2.0	%	1.881	0.214	0.120	*t* test	<0.001
Activated partial thromboplastin time	96	31.4 ± 5.8	34.1 ± 5.1	Second	1.760	−0.198	−0.120	*t* test	0.001
High-density lipoprotein	64	1.4 ± 0.4	1.2 ± 0.4	mmol/L	1.685	0.189	0.116	*t* test	0.002
Urea	78	5.0 ± 2.0	6.5 ± 4.7	mmol/L	1.543	−0.175	−0.092	*t* test	0.003
Globulin	82	27.4 ± 7.7	24.6 ± 4.9	g/L	1.530	0.174	0.093	*t* test	0.004
Serum creatinine	68	64.7 ± 19.4	103.2 ± 126.5	μmol/L	1.528	−0.174	−0.093	*t* test	0.004
Serum sodium	77	141.1 ± 2.6	142.3 ± 3.6	mmol/L	1.483	−0.162	−0.107	*t* test	0.007
Neutrophil count	52	4.4 ± 2.5	5.4 ± 2.8	E + 09/L	1.467	−0.148	−0.055	*t* test	0.012

### Verification by univariate analysis

**Figure 3:**
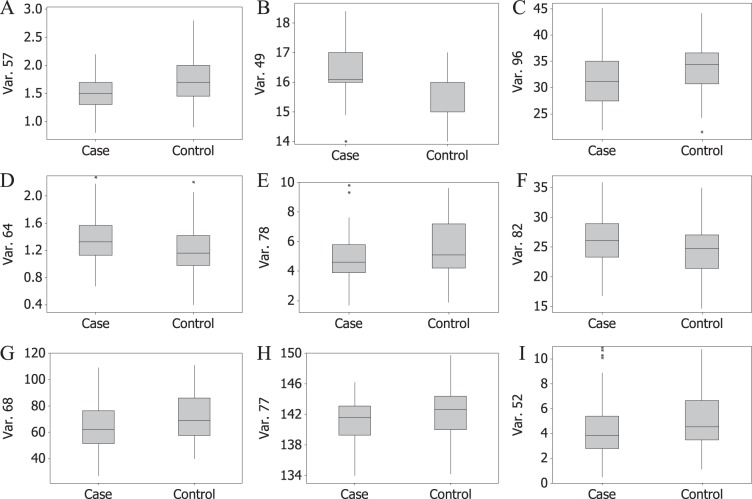
Boxplots of nine continuous variables.

In order to verify the results of PLSR, differences between cases and controls were assessed using univariate data analysis methods. The number of patients who had history of trauma surgery in cases (n = 49) was more than that in controls (n = 17). The boxplots of nine continuous variables are show in Fig. 3. The *chi-square* test was applied for history of trauma surgery and *t* test was applied for nine continuous variables. The univariate data analysis results of ten typical variables are shown in Table 1. As revealed by Table 1, all of the ten variables had significant statistical differences between cases and controls. From an overall perspective, the lower *P* value a variable had, the higher VIP value it had in the PLSR model. The results of univariate data analysis were consistently in agreement with the results of PLSR.

### Development of the software

**Figure 4:**
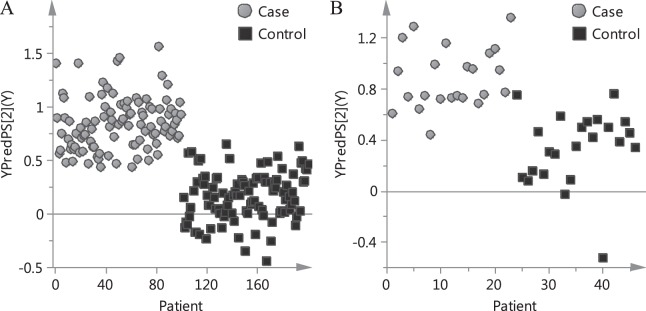
Predicted risk of each patient based on PLSR model. (**A**) Calibration cohort. (**B**) Validation cohort.

**Figure 5:**
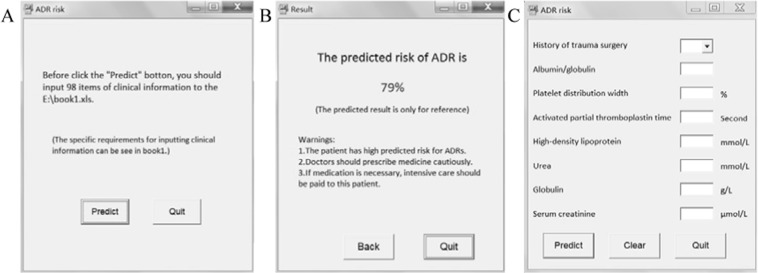
Interfaces of prediction software. (**A**) Initial interface of the software based on PLSR model. (**B**) Result interface. (**C**) Initial interface of the complementary software based on simplified PLSR model.

**Figure 6:**
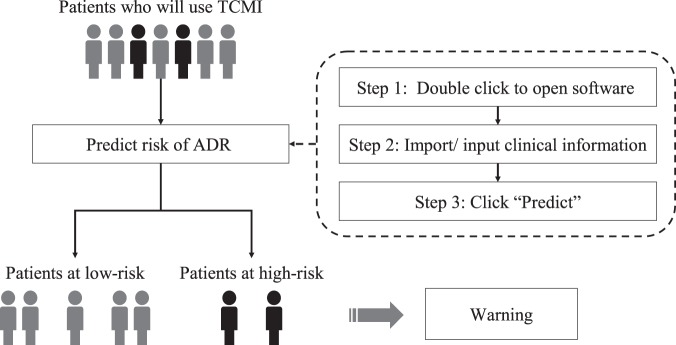
Workflow of recognizing high-risk patient for TCMI-induced ADR.

Based on the PLSR model, the risk of individual patient was predicted. The predicted risk of each patient in calibration cohort is shown in Fig. 4A. On the basis of the established PLSR model, the software was devised. The initial interface of the prediction software based on PLSR model is shown in Fig. 5A. When a clinician intends to prescribe TCMI for a patient, all he/she need to do is the three steps in Fig. 6. The software will substitute the data into the established model and calculate the predicted risk of ADR automatically. The result interface of the software is shown in Fig. 5B. If the predicted risk is greater than the pre-set threshold, the software will give a warning, which alerts clinicians to prescribe TCMI cautiously and reminds nurses to take intensive care toward the patient. In the present study, the threshold was set to 0.6.

### Validation of the method

The effectiveness of the method for new patients was evaluated through its application to external validation cohort. The predicted risk of each patient in validation cohort is shown in Fig. 4B. The patients, whose predicted risks greater than threshold, were recognized as high-risk ones. The recognition accuracy of cases and controls in validation cohort was 95.7% and 91.3%, respectively. The high recognition accuracy indicated that most ADR patients could be correctly recognized.

## Discussion

The regression algorithm is one of the most commonly used statistical technique, which may assume a certain and simple model that links clinical information with ADR. In recent years, many new algorithms which are driven by data and intended to capture the relationships more complex than simple linear form imposed by regression algorithm have been developed to improve the performance of models^12^. Although the new algorithms perform well on the development data, the improvement is limited when tested on new samples. It has been demonstrated that when building predictive models of increasing complexity, the marginal gain from complicated models is typically small compared to the predictive power of the simple models^12^. Moreover, many of the new algorithms are not easy to program and not simple to understand, which limit their applicability. With the aim of providing a convenient approach in routine clinical practice, the PLSR algorithm was finally used to clarify the relationships between clinical information and ADR.

In order to process data from different sources, data fusion strategies including low-level data fusion (LLDF) and high-level data fusion (HLDF) were conducted^13–15^. LLDF is the simplest way to perform data fusion, which is a variable level data fusion method. In this study, the raw basic characteristics and routine examinations were joined at the beginning, and then all variables were balanced to the same scale by unit variance to perform a correct data association. High-level data fusion (HLDF) combines the results obtained from each data set, which is a decision level fusion as the data fusion is done at the final step. In this study, the predicted values obtained from independent basic characteristics and routine examinations were averaged for the final results. The PLSR models based on independent basic characteristics, independent routine examinations and combined clinical information using LLDF and HLDF strategies were compared. Comparison of recognition results of models based on different data sources is outlined in Figure s1A in the Supporting Information. Overall, the model based on LLDF strategy showed a higher accuracy and was applied for the following analysis. As revealed by Fig. 2B, the variables had high VIP values from both basic characteristics and routine examinations, indicating that they both provided valuable information. Distinct overlap of the variables between cases and controls in Fig. 3 suggested that the risk of TCMI-induced ADR was difficult to be predicted by a single variable. From a general point of view, this study was positive and showed the synergy between basic characteristics and routine examination in predicting ADR.

History of trauma surgery was demonstrated to be the most important risk factor for ADR. The patients, who have trauma surgery, may have reduced protein synthesis rate and decreased hepatic drug-metabolizing enzymes activity, leading to increased free drug concentration in plasma, and consequently, give rise to elevated ADR risk. This hypothesis was consistent with our finding that there was higher risk of ADR for patients who had history of trauma surgery. Low albumin/globulin and high globulin were important risk factors for ADR. As indexes to reflect the protein synthesis ability, low albumin and high globulin concentration in plasma indicate malnutrition or hepatobiliary disease. High platelet distribution width and low activated partial thromboplastin time are usually common in patients who had thrombotic diseases. Accordingly, it can be inferred that the patients with malnutrition, hepatobiliary disease or thrombotic diseases had higher risk for ADR. Urea is a metabolite of protein. Low level of urea was an important risk factor, indicating that the patients with deficient protein intake were more prone to have ADR. Renal failure was associated with an increased risk of ADR due to chemical drug^16^. Different from chemical drug, low level of serum creatinine, which is usually an outcome of less muscle quantity caused by malnutrition or chronic wasting disease, was a risk factor for ADR induced by TCMI. Overall, it can be tentatively summarized from the risk factors that the patients had reduced protein synthesis rate, deficient protein intake or chronic wasting disease were more prone to have ADR after using TCMI.

Some other factors have been investigated by post-marketing safety surveillance of TCMI^17–20^. By a descriptive analysis of TCMI-induced ADR cases, the number of women was slightly higher than that of men^19^. In this study, gender had negative regression coefficient (−0.0286) and loading weight (−0.0833) indicating that women were more prone to have ADR. The VIP value of gender (0.854) was low indicating that the effect of gender on ADR was not remarkable. This result was consistently in agreement with the literature. Moreover, it has been reported that the elderly were more prone to have ADR and the differences in specific age groups were significant^19^. In this study, the regression coefficient (0.0277) and loading weight (0.0358) of age was positive, which was consistent with the literature. In addition, there was no significant influence of age on ADR with low VIP value (0.369). The results were not conflict since the elderly were more prone to use TCMI, leading to high rates in both cases and controls. Although the post-marketing studies can explore the epidemiological characteristics of ADR and provide scientific proposals for the clinical application of TCMI, the risk of individual patient can not be predicted under current regulation safety surveillance mode. The potential value of this method in predicting individual’s likelihood of ADR was shown.

In practical use, different thresholds for distinguishing can be applied dynamically according to the practical requirement. The recognition results of PLSR model with different thresholds were compared (Figure s1B in the Supporting Information). A lower threshold means the criterion for recognizing is more relaxed and more patients will be identified as high-risk ones. Therefore, a low threshold can be set if there is adequate medical care personnel in the hospital. Likewise, if a smaller warning rate is expected, the threshold can be set high.

To meet different requirements by achieving a tradeoff among convenience and predictive accuracy, a complementary software product based on fewer variables was devised. The PLSR models based on 8, 30, 50 and 80 variables, which were selected according to the VIP values of the PLSR model, were compared. The recognition results of models based on different number of variables in validation cohort are outlined in Figure s1C in the Supporting Information. Finally, the simplified PLSR model with 8 variables was chosen to devise the complementary software. The initial interface of the complementary software is shown in Fig. 5C. The result interface is shown in Fig. 5B, which is the same with the software based on PLSR model. As it was expected, there were higher predictive abilities of models based on more variables. However, the complementary software was more intuitive and could be applied to assist preliminary therapeutic decisions by facilitating prescreening of patients with 87.0% accuracy of cases and 82.6% accuracy of controls in external validation cohort. The two software products can be selected according to the practical requirement. With the proper application of the software, some of the ADR may be prospectively preventable.

On account of the occurrence rate, strict inclusion and exclusion criteria, there were limited number of samples in this study. Firstly, all the cases in our study are population-based real-world practice. Nevertheless, the occurrence rate of suspected ADR was lower than one-in-a-thousand. Secondly, we tried to reduce bias by strict inclusion criteria. Only when 6 researchers in two teams met to reach a consensus decision that the ADR had definite or probable relation with the use of TCMI, the cases were considered to be eligible for study enrollment. Thirdly, we tried to increase the power of this model by strict exclusion criteria. The cases were excluded if the routine examinations were not taken at baseline, or if there was less than 3 cases of a TCMI occurred over the study period. Finally, 123 cases and 123 controls were involved. Although the number was limited, all the cases in our study were non-interventional clinical events, which reflected real-world practice. In addition, the accuracy and robustness of this method was demonstrated with new patients in external validation cohort. Nevertheless, the method still needs improvement and validation through larger-scale and longer-term clinical practice.

The main limitation of this study was the ignorance of manifestations, seriousness of ADR and sorts of TCMI because of limited number of samples in each category. However, the differences between cases and controls were demonstrated. On the basis of this study, more studies will be carried out to clarify the characteristics in different categories by including more samples in our further works.

## Methods

### Study design and population

This population-based study was conducted among adult hospitalized patients (aged ≥18 years) at two hospitals in China. One is Tongde hospital of Zhejiang province, which is an integrated Chinese and western medicine hospital with a total capacity of 1800 licensed beds. The other is Zhejiang province hospital of traditional Chinese medicine, which is a 2000-beds traditional Chinese medicine hospital. According to the requirements of “National ADR Reporting and Monitoring Management Measures”, the information of ADR was routinely recorded to fill the “ADR Monitoring Information Form” if suspected ADR was observed. The information of ADR for patients who used TCMI was retrospectively extracted from the ADR monitoring system and then the determination of whether an ADR contributed to TCMI was conducted using the relevance evaluation method of the national ADR monitoring center (Table s4 in the Supporting Information) by two research teams independently. Each team comprised a senior nurse, clinical pharmacist and clinician. After detailed reviewing and comprehensive analyzing the information of patient, medication and ADR by the senior nurse, the ADR was preliminarily classified into six grades, including definite, probable, possible, unlikely, pending and unassessable. The classification was then confirmed by the clinical pharmacist and clinician. Meanwhile, a blind and independent assessment was undertaken by three researches in the other team. Only all the researchers met to reach a consensus decision that the ADR had definite or probable relation with the use of TCMI, the case was incorporated into the study.

The study used a nested case-control strategy^21–24^, which nested within a cohort of the confirmed ADR cases. Controls were randomly selected from the patients who had no ADR in the same hospital to match each ADR case. The cases and matched controls used the TCMI with same batch at the same day to reduce the effects of drugs. The dosage and frequency were also controlled to eliminate the influence of medication. Accordingly, the cases and the same number of controls were involved.

### Data management

In this study, the basic characteristics and routine examinations at baseline were retrospectively extracted from electronic medical records system. Totally 98 items were collected for each case and encoded to build *X* matrix. The categories, names and descriptions of clinical information are outlined in Table s5 in the Supporting Information. The grouping information was encoded as *Y* matrix through assigning case to 1 and assigning control to 0.

### Statistical methods

The patients from January 2013 to December 2017 were utilized as calibration cohort to establish the PLSR models and devise the software. An external validation was carried out based on the patients from January 2018 to December 2018. The patients with predicted ADR-risks greater than pre-set threshold were recognized as high-risk ones. The recognition accuracy of cases and controls was conducted for evaluating the predictive capacity. PLSR was performed using SIMCA-P + (Umetrics, Sweden, version 13.0). *Chi-square* test and *t* test were applied to verify the results of PLSR, which were performed using MINITAB (Minitab Inc., USA, version 14.1). Visual Basic (Microsoft, USA, version 6.0) was used as integrated development environment.

### Ethics, consent and permissions

The study was approved by the ethics committee of Tongde hospital of Zhejiang province and Zhejiang province hospital of traditional Chinese medicine. The study was performed in accordance with the reporting guidelines and Helsinki declaration. On the basis of the “Methods for ethical review of biomedical research involving human beings” (chapter 4 article 39) in the order NO.11 of the national health and family planning commission of China, which was issued in October 2016, the informed consent can be waived by using human data if it is impossible to find the subject, and the research project does not involve personal privacy and business interests. Accordingly, informed consent was waived due to retrospective design of this study after being approved by the ethics committee. To protect the privacy of patients, the detailed data from the hospital information system was confidential.

## Supplementary information

Supplementary Information
