# Supplementary material for: Identification of high-risk patients for ADR induced by traditional Chinese medicine injection: a nested case-control study
Source: Sci Rep. 2019 Nov 13;9:16721. doi: 10.1038/s41598-019-53267-2 (PMC6853959; doi:10.1038/s41598-019-53267-2)
Supplement: Supplementary file 1 — Supplementary Information [file 41598_2019_53267_MOESM1_ESM.pdf]

Identification of high-risk patients for ADR induced by traditional Chinese medicine injection: a nested case-control study

Cheng Jiang,[1,#] Jie Shen,[2,#] Dan Shou,[3] Nani Wang,[3] Jing Jing,[4] Guodi Zhang,[4] Jing Gu,[4] Yunlong Tian,[5] Caihua Sun,[2] Jiaqi He,[2] Jiaqi Ma,[6] Xiaojun Wang,[1,*] Gonghua Li,[1,**]

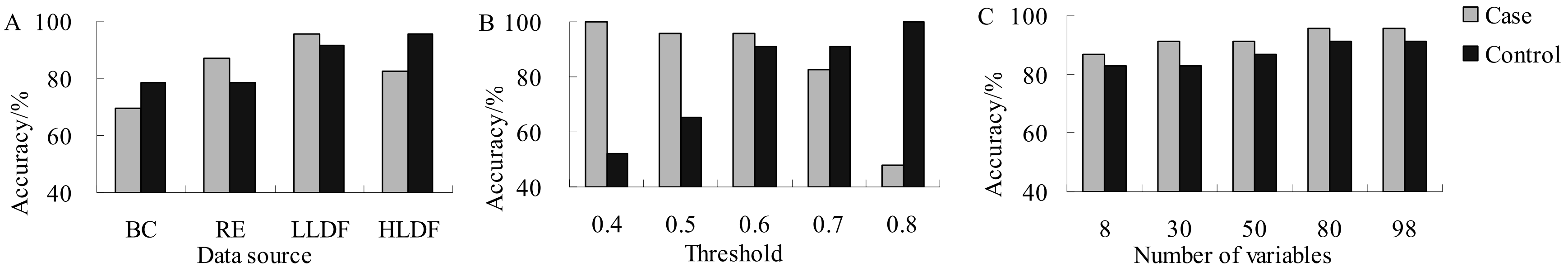

Figure s1. Recognition results of models in validation cohort. **(A)** Models based on different data sources. (BC: Basic characteristic; RE: Routine examination) **(B)** Models with different thresholds. **(C)** Models based on different number of variables.

TCMI names	Number	Herbal species	Major active compounds	References
Shenmai injection	82	*Radix Ginseng Rubra*	Ginsenoside Re, Rg1, Rf, et al.	[1-4]
		Radix Ophiopogonis	Ophiopogonin D, ophiopogonanone A, 2'-Hydroxyophiopogonone A, et al.	
Kudiezi injection	10	*Ixeridium Sonchifolium*	Sonchifoliasolide M, sonchifoliactone C, ixerisoside C, et al.	[5-7]
Shuxuening injection	8	*Ginkgo Folium*	Rutin, isoquercitrin, kaempferol, et al.	[8-11]
Danhong injection	7	*Radix Salviae*	Protocatechuic aldehyde, tanshinol, salvianolic acid A, et al.	[12-14]
		Carthami Flos	Syringin, quercimeritrin, caffeic acid, et al.	
Xiaoaiping injection	7	*Marsdenia Tenacissima*	Tenacigenin A, B, tenacissoside F, et al.	[15]
Kangai injection	6	*Hedysarum Multijugum*	Astragaloside IV, calycosin, et al.	[16]
		Panax Ginseng	Ginsenoside Re, Rg1, Rf, et al.	
		Sophorae Flavescentis Radix	Oxymatrine	
Kanglaite injection	3	*Coicis Semen*	Triolein, coixenolide, coixol, et al.	[17]

Table s1. Herbal species and major active compounds of each TCMI

Systems / organizations	Total number of cases*	Main manifestations (number of cases*)
Systemic injury	56	Hot flush (35), shiver (11), fever (6), acratia (3), chest pain (1)
Respiratory system damage	54	Chest tightness (33), dyspnea (21)
Skin lesion	37	Erythra(20), pruritus (13), hypethidorosis (4)
Gastrointestinal system damage	21	Sicchasia (11), vomit (8), abdominal pain (2)
Nervous system damage	8	Giddy (5), headache (2), paresthesia (1)
Cardiovascular system damage	5	Hypertension (3), phlebitis (2)

Table s2. Main ADR manifestations in included cases

* There may be several manifestations occurred simultaneously in one ADR case.

Classification of seriousness		Number of cases	Constituent ratio/%
Class I	ADR are deadly or life-threatening.	4	3.25
Class II	Patients have pathologic and physiologic changes.	3	2.44
Class III	Patients cannot endure the ADR and must be given a lower dose or withdrawn from drugs.	20	16.26
Class IV	Patients can endure the ADR.	96	78.05

Table s3. ADR seriousness in included cases

Classification of relevance	Point 1*	Point 2+	Point 3&	Point 4#	Point 5$
Definite	Yes	Yes	Yes	Yes	Yes
Probable	Yes	Yes	Yes	Uncertainty	Yes
Possible	Yes	Yes	Uncertainty	Uncertainty	Uncertainty
Unlikely	No	No	Uncertainty	Uncertainty	Uncertainty
Pending	There are missing contents. The evaluation can be completed when the supplementary specifications are provided.				
Unassessable	Many items are unavailable. The relationship is unassessable since the missing items can not be supplemented.				

Table s4. Relevance evaluation method of the national ADR monitoring center

*: The temporal relation between medication and ADR's occurrence was reasonable;

+: The clinical symptoms were coincided with the known ADR profile;

&: The ADR was relieved or vanished after decreasing dosage or drug withdrawal;

#: The ADR occurred again when drug was re-administered;

$: The probability of ADR caused by concomitant medications, disease progression or other treatment could be excluded.

Variable categories		Variable NO.: variable names (variable descriptions)
Basic characteristic	Demographic	1: Gender (male: 1, female: 0); 2: Age (year); 3: Body weight (kg); 4: Height (cm);
	Comorbiditie	5: Hypertension (yes: 1, no: 0); 6: Diabetes (yes: 1, no: 0); 7: Hepatitis (yes: 1, no: 0); 8: Tuberculosis (yes: 1, no: 0);
	Previous history	9: History of trauma surgery (yes: 1, no: 0); 10: History of drug allergy (yes: 1, no: 0); 11: History of food allergy (yes: 1, no: 0); 12: History of blood transfusion (yes: 1, no: 0);
Routine examination	Urine flow analysis	13: Urinary sediment cast (/μL); 14: Urinary sediment conductivity (mS/cm); 15: Urinary sediment epithelial cell (/μL); 16: Urinary sediment mucus (/μL); 17: Urinary sediment path cast (/μL); 18: Urine pH; 19: Urinary sediment red blood cell (/μL); 20: Urinary sediment small round cell (/μL); 21: Urinary sediment white blood cell (/μL); 22: Urinary sediment yeast-like fungi (/μL); 23: Urine white blood cell (negative: 0, +: 1, ++: 2); 24: Urine specific gravity; 25: Urine bilirubin (negative: 0, +: 1, ++: 2); 26: Urine protein (negative: 0, +: 1, ++: 2); 27: Urobilinogen (negative: 0, +: 1, ++: 2); 28: Urine glucose (negative: 0, +: 1, ++: 2); 29: Urine acetone bodies (negative: 0, +: 1, ++: 2); 30: Urine nitrous acid (negative: 0, +: 1, ++: 2); 31: Hematuria (negative: 0, +: 1, ++: 2);
	Blood routine examination	32: White blood cell count (E+09/L); 33: Monocyte count (E+09/L); 34: Monocytes ratio (%); 35: Red blood cell distribution width (%); 36: Red blood cell count (E+12/L); 37: Red blood cell hematocrit (%); 38: Lymphocyte count (E+09/L); 39: Lymphocyte ratio (%); 40: Mean corpuscular volume (fL); 41: Mean corpuscular hemoglobin (pg); 42: Mean corpuscular hemoglobin concentration (g/L); 43: Mean platelet volume (fL); 44: Basophilic granulocyte count (E+09/L); 45: Basophilic granulocyte ratio (%); 46: Eosinophilic granulocyte count (E+09/L); 47: Eosinophilic granulocyte ratio (%); 48: Hemoglobin (g/L); 49: Platelet distribution width (%); 50: Blood platelet count (E+09/L); 51: Thrombocytocrit (%); 52: Neutrophil count (E+09/L); 53: Neutrophil ratio (%);
	Biochemical series examination	54: Aspertate aminotransferase/alanine aminotransferase ; 55: b-hydroxybutyric acid (mmol/L); 56: Albumin (g/L); 57: Albumin/globulin; 58: High-sensitivity C-reactive protein (mg/L); 59: Cholinesterase (U/L); 60: Low density lipoprotein (mmol/L); 61: Amylase (U/L); 62: Glycylproline dipeptidyl aminopeptidase (U/L); 63: Triglyceride (mmol/L); 64: High-density lipoprotein (mmol/L); 65: Glutamyl transpeptidase (U/L); 66: Alanine aminotransferase (U/L); 67: Aspertate aminotransferase (U/L); 68: Serum creatinine (μmol/L); 69: Creatine kinase isoenzyme (U/L); 70: Potassium (mmol/L); 71: Indirect bilirubin (μmol/L); 72: Alkaline phosphatase (U/L); 73: Phosphorus (mmol/L); 74: Creatine phosphate kinase (U/L); 75: Chlorine (mmol/L); 76: Magnesium (mmol/L); 77: Sodium (mmol/L); 78: Urea (mmol/L); 79: Uric acid (μmol/L); 80: Glucose (mmol/L); 81: Hydroxybutyrate dehydrogenase (U/L); 82: Globulin (g/L); 83: Lactic dehydrogenase (U/L); 84: Bicarbonate (mmol/L); 85: Homocysteine (μmol/L); 86: Adenosine deaminase (U/L); 87: Apolipoprotein A (g/L); 88: Apolipoprotein B (g/L); 89: Direct bilirubin (μmol/L); 90: Total cholesterol (mmol/L); 91: Total bilirubin (μmol/L); 92: Total bile acid (μmol/L); 93: Total protein (g/L); 94: Total calcium (mmol/L);
	Coagulation function examination	95: International normalized ratio; 96: Activated partial thromboplastin time (second); 97: Prothrombin time (second); 98: Fibrinogen (g/L)

Table s5. Categories, names and descriptions of clinical information

References:

1. Fang, X., Wang, Y. & Cheng, Y. LC/MS fingerprinting of Shenmai injection: a novel approach to quality control of herbal medicines. *J Pharmaceut Biomed* **40**, 591-597 (2006).
2. Yu, J. *et al*. Simultaneous determination and pharmacokinetics of eight ginsenosides by LC-MS/MS after intravenously infusion of 'SHENMAI' injection in dogs. *Pak J Pharm Sci* **30**, 421-427 (2017).
3. Yu, K., Ma, Y., Shao, Q., Qu, H. & Cheng, Y. Simultaneously determination of five ginsenosides in rabbit plasma using solid-phase extraction and HPLC/MS technique after intravenous administration of 'SHENMAI' injection. *J Pharmaceut Biomed* **44**, 532-539 (2007).
4. Xia, C. *et al*. Simultaneous determination of ginsenoside Rg1, Re, Rd, Rb1 and ophiopogonin D in rat plasma by liquid chromatography/electrospray ionization mass spectrometric method and its application to pharmacokinetic study of 'SHENMAI' injection. *J Chromatogr B* **862**, 72-78 (2008).
5. Shi, P. *et al*. Simultaneous Determination of Five Phenolic Acids and Four Flavonoid Glycosides in Rat Plasma Using HPLC-MS/MS and Its Application to a Pharmacokinetic Study after a Single Intravenous Administration of Kudiezi Injection. *Molecules* **24**, 64 (2018).
6. Liu, R. *et al*. Rapid screening and identification of sesquiterpene lactones in Kudiezi injection based on high-performance liquid chromatography coupled with linear ion trap-orbitrap mass spectrometry. *Chin J Nat Medicines* **16**, 150-160 (2018).
7. Yin, R. *et al*. UFLC-MS/MS method for simultaneous determination of luteolin-7-O-gentiobioside, luteolin-7-O-β-D-glucoside and luteolin-7-O-β-D-glucuronide in beagle dog plasma and its application to a pharmacokinetic study after administration of traditional Chinese medicinal preparation: Kudiezi injection. *J Pharmaceut Biomed* **72**, 127-133 (2013).
8. Ma, H., Li, J., An, M., Gao, X. & Chang, Y. A powerful on line ABTS-CE-DAD method to screen and quantify major antioxidants for quality control of Shuxuening Injection. *Sci Rep-Uk* **8**, 5441 (2018).
9. Lin, S. *et al*. Development and Validation of an Analytical Method for the Determination of Flavonol Glycosides in Ginkgo Leaves and ShuXueNing Injections by a Single Marker. *J Chromatogr Sci* **54**, 1041-1049 (2016).
10. Yu, S. *et al*. Integrated liquid chromatography-mass spectrometry and nuclear magnetic resonance spectra for the comprehensive characterization of various components in the Shuxuening injection. *J Chromatogr A* **1559**, 125-135 (2019).
11. Niu, Y. *et al*. Development of an on-line analytical platform to screen haptens in shuxuening injection based on high-performance liquid chromatography coupled with ion-trap multistage mass spectrometry and human serum albumin fluorescence detection. *J Chromatogr A* **1598**, 232-241 (2019).
12. Li, X. *et al*. Simultaneous determination of eight Danshen polyphenols in rat plasma and its application to a comparative pharmacokinetic study of DanHong injection and Danshen injection. *J Sep Sci* **40**, 1470-1481 (2017).
13. Liu, H. *et al*. Characterization of in vivo antioxidant constituents and dual-standard quality assessment of Danhong injection. *Biomed Chromatogr* **27**, 655-663 (2013).
14. Qi, Y. *et al*. Identification of a Quality Marker (Q-Marker) of Danhong Injection by the Zebrafish Thrombosis Model. *Molecules* **22**, 1443 (2017).
15. Gong, X. *et al*. Fingerprint Analysis of Xiaoaiping Injection and Simultaneous Determination of Its Seven Active Steroidal Components in Xiaoaiping Injection by HPLC-ELSD. *Chin J Exper Trad Med Form* **22**, 68-72 (2016).
16. Guo, Y. & Liu, Q. The Studies of Chemical Constituents of Kang'ai Injection. *Pharm Tod* **29**, 6-10 (2019).
17. Wang, B. Network Pharmacology Study of Anticancer Mechanism of Three Main Components in Kanglaite Injection. *Chin J Mod Appl Pharm* **36**, 58-63 (2019).
